# Supplementary material for: Chromothripsis during telomere crisis is independent of NHEJ, and consistent with a replicative origin
Source: Genome Res. 2019 May;29(5):737–49. doi: 10.1101/gr.240705.118 (PMC6499312; doi:10.1101/gr.240705.118)
Supplement: Supplemental Material [file supp_gr.240705.118_Supplemental_file_1.zip › contigs/annotated_contigs/DB87/contig.2.DB87_length_696_mean_cov_23.349137931.docx]

**DB87_length_696_mean_cov_23.349137931**

```
TGATGATGTTGACAAGAGCCAGGTCAATAGAGTGGGACACAGTAGCCTTTTGAGAATGAATTGAGGAGAACCTGGTGCTACCACTCACG
 >chr7:159044366-159044660 - E=4e-165
GTCATGGTGTGGAACAGGAAAAACATGGGGTTTCAAATGTGCAGGTCTTCGTGTTCATTACAAAGAAAGACCAACCATATTTTTAAAAT

ATTTTGCTGTATTTCGTAGCTTAAAATATTGAGAAAAAAATGATCTGATTTCCTAAAGAGACCAGGTGTTACAATGGAAGACCTACTTT

CTAGGAAATAAAATTCTCACATGAATC|TGTCTTATTTAGCACCTTGTCAATGATTTGTACTGTTACTCTAGATATACAAGAAATATTT
                             >chr7:159056215-159056617 - E=4e-230
TCTGTCATTGAAGTTCAATAACTTGAACAGCATGTATTTTGGTGTTGAGCATTCTGTATCAACTATCCCTCAAAGGGCATATACTTTTT

AAAATTATAGATTAAATTATTCATTTTGGGAAAATTCTATATTATAAACATCTTCCCTCTTTCCTTCACTGATTTTTCTACTTTAGGGA

TGCCAATTATTTTTATTTTGGACCACCCTTGTCTTTCTTCATCTTTCTCTCTTTCTTTTCTTTCTTTCTTTCTCTCTCCCTCTTTCTTC

TTTTTGATTTGATCTTCTCTTTCCAAAAGTAGTCACTGTGATTATGTCAAAATTTTTATTTTGGTCAATAATTA
```
